# Supplementary material for: Machine Learning Accelerates Crystallization for Structure Determination
Source: Angew Chem Int Ed Engl. 2026 May 4;65(25):e1218503. doi: 10.1002/anie.1218503 (PMC13266958; doi:10.1002/anie.1218503)

checkCIF/PLATON report

Structure factors have been supplied for datablock(s) lcz095

THIS REPORT IS FOR GUIDANCE ONLY. IF USED AS PART OF A REVIEW PROCEDURE FOR PUBLICATION, IT SHOULD NOT REPLACE THE EXPERTISE OF AN EXPERIENCED CRYSTALLOGRAPHIC REFEREE.

No syntax errors found.   CIF dictionary   Interpreting this report

Datablock: lcz095

Bond precision:	C-C = 0.0119 A		Wavelength=1.54184
Cell:	a=23.0121(4)	b=12.9825(2)	c=12.1703(2)
	alpha=90	beta=94.380(1)	gamma=90
Temperature:	100 K		

	Calculated	Reported
Volume	3625.31(10)	3625.31(10)
Space group	C 2/c	C 1 2/c 1
Hall group	-C 2yc	-C 2yc
Moiety formula	C15 H3 Ag3 F18 N6, C16 H16	C15 H3 Ag3 F18 N6, C16 H16
Sum formula	C31 H19 Ag3 F18 N6	C31 H19 Ag3 F18 N6
Mr	1141.13	1141.13
Dx,g cm-3	2.091	2.091
Z	4	4
Mu (mm-1)	14.057	14.057
F000	2200.0	2200.0
F000'	2210.23	
h,k,lmax	29,16,15	29,16,15
Nref	3892	3742
Tmin,Tmax	0.104,0.140	0.447,1.000
Tmin'	0.046	

Correction method= # Reported T Limits: Tmin=0.447 Tmax=1.000
AbsCorr = MULTI-SCAN

Data completeness= 0.961 Theta(max)= 78.364

R(reflections)= 0.0550(3337) wR2(reflections)= 0.1680(3742)

S = 1.108 Npar= 264

The following ALERTS were generated. Each ALERT has the format
test-name_ALERT_alert-type_alert-level.
Click on the hyperlinks for more details of the test.

Alert level C

```
PLAT213_ALERT_2_C Atom F00A           has ADP max/min Ratio .....        3.5 prolat
PLAT213_ALERT_2_C Atom F00C           has ADP max/min Ratio .....        3.2 prolat
PLAT213_ALERT_2_C Atom F00K           has ADP max/min Ratio .....        3.3 prolat
PLAT250_ALERT_2_C Large U3/U1 Ratio for <U(i,j)> Tensor(Resd    2)       2.5 Note
PLAT342_ALERT_3_C Low Bond Precision on  C-C Bonds ..............    0.01187 Ang.
PLAT911_ALERT_3_C Missing FCF Refl Between Thmin & STh/L=    0.600         3 Report
               14 12  5, -20  4 10,  -6  0 10,
PLAT971_ALERT_2_C Check Calcd Resid. Dens.  0.95Ang From C00U           2.21 eA-3
PLAT971_ALERT_2_C Check Calcd Resid. Dens.  0.97Ang From Ag01           1.83 eA-3
PLAT971_ALERT_2_C Check Calcd Resid. Dens.  0.97Ang From Ag01           1.64 eA-3
```

Alert level G

```
PLAT003_ALERT_2_G Number of Uiso or U(i,j)  Restrained non-H-Atoms         2 Report
PLAT072_ALERT_2_G SHELXL First  Parameter in WGHT  Unusually Large      0.12 Report
PLAT177_ALERT_4_G The CIF-Embedded .res File Contains DELU Records         1 Report
PLAT192_ALERT_3_G A Non-default DELU Restraint Value for SecondPar    0.0200 Report
PLAT232_ALERT_2_G Hirshfeld Test Diff (M-X)  Ag01     --N004     .       5.5 s.u.
PLAT242_ALERT_2_G Low    'MainMol' Ueq as Compared to Neighbors of      C00I Check
PLAT242_ALERT_2_G Low    'MainMol' Ueq as Compared to Neighbors of      C00O Check
PLAT242_ALERT_2_G Low    'MainMol' Ueq as Compared to Neighbors of      C00T Check
PLAT434_ALERT_2_G Short Inter HL..HL Contact F00A     ..F00K     .      2.78 Ang.
                                     3/2-x,1/2+y,1/2-z  =      4_655 Check
PLAT720_ALERT_4_G Number of Unusual/Non-Standard Labels ..........        40 Note
              Ag01    Ag02    F003    N004    F005    N006    N007    F008
              F009    F00A    F00B    F00C    C00D    C00E    H00E    F00F
              C00G    H00G    C00H    C00I    C00J    F00K    C00L    C00M
              H00M    C00N    H00N    C00O    C00P    H00P    C00Q    H00A
              H00B    H00C    C00R    H00R    C00S    C00T    C00U    H00U
PLAT860_ALERT_3_G Number of Least-Squares Restraints ............          1 Note
PLAT883_ALERT_1_G Absent Datum for _atom_sites_solution_primary ..     Please Do !
PLAT912_ALERT_4_G Missing # of FCF Reflections Above STh/L=  0.600       147 Note
PLAT941_ALERT_3_G Average HKL Measurement Multiplicity ..........        2.9 Low
PLAT969_ALERT_5_G The 'Henn et al.' R-Factor-gap value ..........      3.654 Note
              Predicted wR2: Based on SigI**2  4.60 or SHELX Weight 15.16
PLAT978_ALERT_2_G Number C-C Bonds with Positive Residual Density.         0 Info
```

---

0 **ALERT level A** = Most likely a serious problem - resolve or explain
0 **ALERT level B** = A potentially serious problem, consider carefully
9 **ALERT level C** = Check. Ensure it is not caused by an omission or oversight
16 **ALERT level G** = General information/check it is not something unexpected

```
 1 ALERT type 1 CIF construction/syntax error, inconsistent or missing data
15 ALERT type 2 Indicator that the structure model may be wrong or deficient
 5 ALERT type 3 Indicator that the structure quality may be low
 3 ALERT type 4 Improvement, methodology, query or suggestion
 1 ALERT type 5 Informative message, check
```

Validation response form

Please find below a validation response form (VRF) that can be filled in and pasted into your CIF.

```
# start Validation Reply Form
_vrf_PLAT213_lcz095
;
PROBLEM: Atom F00A            has ADP max/min Ratio .....        3.5 prolat
RESPONSE: ...
;
_vrf_PLAT250_lcz095
;
PROBLEM: Large U3/U1 Ratio for <U(i,j)> Tensor(Resd    2)        2.5 Note
RESPONSE: ...
;
_vrf_PLAT342_lcz095
;
PROBLEM: Low Bond Precision on  C-C Bonds ..............    0.01187 Ang.
RESPONSE: ...
;
_vrf_PLAT911_lcz095
;
PROBLEM: Missing FCF Refl Between Thmin & STh/L=    0.600          3 Report
RESPONSE: ...
;
_vrf_PLAT971_lcz095
;
PROBLEM: Check Calcd Resid. Dens.  0.95Ang From C00U           2.21 eA-3
RESPONSE: ...
;
# end Validation Reply Form
```

It is advisable to attempt to resolve as many as possible of the alerts in all categories. Often the minor alerts point to easily fixed oversights, errors and omissions in your CIF or refinement strategy, so attention to these fine details can be worthwhile. It is up to the individual to critically assess their own results and, if necessary, seek expert advice.

PLATON version of 17/09/2025; check.def file version of 17/09/2025

---

duplicate check

No duplication found

Datablock lcz095 - ellipsoid plot

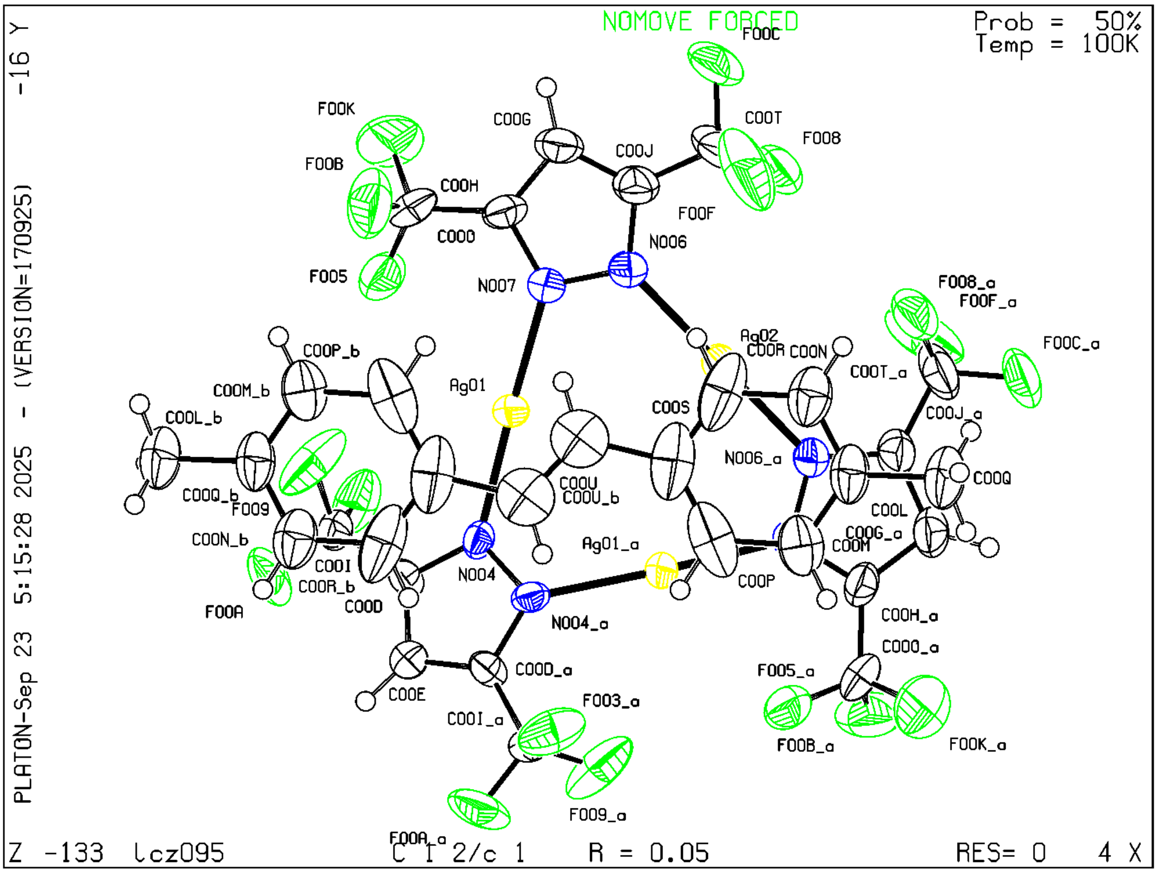

Supplement: Supplementary file 2 — Supporting File 2: anie72462‐sup‐0002‐DataFile.zip. [file ANIE-65-e1218503-s002.zip › supplementary_materials_crystallographic_data submit.zip!/cif_and_checkcif(1-114)/Ag3Pz3_95/Ag3Pz3_95_checkcif.pdf]
